# Whole-genome analysis of human papillomavirus genotypes 52 and 58 isolated from Japanese women with cervical intraepithelial neoplasia and invasive cervical cancer

**DOI:** 10.1186/s13027-017-0155-4

**Published:** 2017-08-04

**Authors:** Yuri Tenjimbayashi, Mamiko Onuki, Yusuke Hirose, Seiichiro Mori, Yoshiyuki Ishii, Takamasa Takeuchi, Nobutaka Tasaka, Toyomi Satoh, Tohru Morisada, Takashi Iwata, Shingo Miyamoto, Koji Matsumoto, Akihiko Sekizawa, Iwao Kukimoto

**Affiliations:** 1 Department of Obstetrics and Gynecology, Showa University School of Medicine, Tokyo, Japan; 2 Pathogen Genomics Center, National Institute of Infectious Diseases, 4-7-1 Gakuen, Musashi-murayama, Tokyo, 208-0011 Japan; 3 Department of Obstetrics and Gynecology, Faculty of Medicine, University of Tsukuba, Tsukuba, Japan; 4 Department of Obstetrics and Gynecology, Keio University School of Medicine, Tokyo, Japan

**Keywords:** Human papillomavirus, HPV52/58, Cervical cancer, Variant, SNPs

## Abstract

**Background:**

Human papillomavirus genotypes 52 and 58 (HPV52/58) are frequently detected in patients with cervical intraepithelial neoplasia (CIN) and invasive cervical cancer (ICC) in East Asian countries including Japan. As with other HPV genotypes, HPV52/58 consist of multiple lineages of genetic variants harboring less than 10% differences between complete genome sequences of the same HPV genotype. However, site variations of nucleotide and amino acid sequences across the viral whole-genome have not been fully examined for HPV52/58. The aim of this study was to investigate genetic variations of HPV52/58 prevalent among Japanese women by analyzing the viral whole-genome sequences.

**Methods:**

The entire genomic region of HPV52/58 was amplified by long-range PCR with total cellular DNA extracted from cervical exfoliated cells isolated from Japanese patients with CIN or ICC. The amplified DNA was subjected to next generation sequencing to determine the complete viral genome sequences. Phylogenetic analyses were performed with the whole-genome sequences to assign variant lineages/sublineages to the HPV52/58 isolates. The variability in amino acid sequences of viral proteins was assessed by calculating the Shannon entropy scores at individual amino acid positions of HPV proteins.

**Results:**

Among 52 isolates of HPV52 (CIN1, *n* = 20; CIN2/3, *n* = 21; ICC, *n* = 11), 50 isolates belonged to lineage B (sublineage B2) and two isolates belonged to lineage A (sublineage A1). Among 48 isolates of HPV58 (CIN1, *n* = 21; CIN2/3, *n* = 19; ICC, *n* = 8), 47 isolates belonged to lineage A (sublineages A1/A2/A3) and one isolate belonged to lineage C. Single nucleotide polymorphisms specific for individual variant lineages were determined throughout the viral genome based on multiple sequence alignments of the Japanese HPV52/58 isolates and reference HPV52/58 genomes. Entropy analyses revealed that the E1 protein was relatively variable among the HPV52 isolates, whereas the E7, E4, and L2 proteins showed some variations among the HPV58 isolates.

**Conclusions:**

Among the HPV52/58-positive specimens from Japanese women with CIN/ICC, the variant distributions were strongly biased toward lineage B for HPV52 and lineage A for HPV58 across histological categories. Different patterns of amino acid variations were observed in HPV52 and HPV58 across the viral whole-genome.

**Electronic supplementary material:**

The online version of this article (doi:10.1186/s13027-017-0155-4) contains supplementary material, which is available to authorized users.

## Background

Human papillomaviruses (HPVs) constitute a large family of small DNA viruses, having a circular double-stranded DNA genome of approximately 8000 base pairs [1]. Their genomes share the same genomic organization, and are composed of at least eight coding regions (early genes: E1, E2, E4, E5, E6, and E7; late genes: L1 and L2) and two non-coding regions, including the long control region (LCR). So far, more than 200 different genotypes of HPV have been identified as showing more than 10% differences of the L1 nucleotide sequence in relation to other genotypes [2]. At least 13 genotypes (HPV16, 18, 31, 33, 35, 39, 45, 51, 52, 56, 58, 59, and 68) [3], referred to as “high-risk” HPVs, are recognized as the causative agents of cervical cancer and many other cancers, including vaginal, vulvar, penile, anal, and oropharyngeal cancers [4]. High-risk HPVs preferentially infect basal epithelial cells and induce hyper-proliferative lesions that are clinically manifested as cervical intraepithelial neoplasia grade 1 (CIN1) in the cervix. The majority of such infections are cleared by the host immune system within a few years, and only a small proportion persist and progress further to CIN grade 2 or 3 (CIN2/3). These high-grade lesions eventually develop into invasive cervical cancer (ICC) by accumulating host genetic alternations after years of persistent infection [5].

Among the high-risk HPVs, HPV16 and HPV18 are the most and second most prevalent genotypes in ICC, respectively, in total accounting for about 70% of ICC cases worldwide [6]. Although the high prevalence of HPV16/18 in ICC is common throughout the world, the distribution of other high-risk HPVs in the remaining fraction of ICC shows some region-specific variations [7]. In particular, in East Asian countries including China, Taiwan, South Korea and Japan, HPV52 and HPV58 infections are more prevalent compared to European, North American and African regions [8, 9]. In Japan, HPV52 and HPV58 ranked the second and third, respectively, in CIN2/3 cases, and ranked the third and fourth in ICC cases, accounting for 8–9% and 3–5% of ICC, respectively [10, 11].

HPV genomes with less than 10% differences in their L1 sequences, recognized as intra-type variants, constitutes an additional level of HPV genetic complexity. Based on complete viral genome sequences, intra-type variants are phylogenetically classified into different lineages and sublineages, which are defined as containing 1.0–10.0% and 0.5–1.0% nucleotide variations, respectively [12]. As such, HPV52 is classified into four variant lineages (A, B, C, and D) and seven sublineages (A1, A2, B1, B2, C1, C2, and D), whereas HPV58 consists of four variant lineages (A, B, C, and D) and eight sublineages (A1, A2, A3, B1, B2, C, D1, and D2) [12].

Many lines of evidence attribute a higher risk of progression to ICC to some distinct variant lineages of high-risk HPVs. Intriguingly, recent large-scale studies revealed that different HPV16 variant sublineages were associated with different risks for cervical squamous cell carcinoma or adenocarcinoma [13–15]. A study investigating the worldwide distribution of HPV52 variants also suggested that lineage B (in particular sublineage B2) posed a higher risk for cervical cancer development among the variant lineages [16]. In a study from Taiwan on HPV52 variants, however, lineage C infection posed a higher risk for CIN3/ICC compared to lineage B [17]. Regarding HPV58 variants and their disease association, lineage A was suggested to be more closely associated with persistent infection compared to other lineages [18], and subsequent studies reported that sublineage A1 or A3 might be associated with a risk for CIN3/ICC [17, 19].

While HPV52/58 infections are common in East Asia, comprehensive surveys have not been conducted regarding their variant distributions in Japan. Determining the complete viral genome sequence is the most reliable and accurate procedure to assign HPV variant lineages/sublineages compared with utilizing only limited sequences in the HPV genome, which was generally done in previous studies on HPV52/58 variant classification [17, 20–22]. This study thus aimed to collect the whole-genome sequences of HPV52/58 from Japanese women with CIN/ICC through next generation sequencing techniques and perform in-depth analyses of genetic variations of these particular HPVs.

## Methods

### Study samples

Cervical exfoliated cells were collected in ThinPrep media (Hologic, Bedford, MA) using a cytoblush from Japanese patients diagnosed with CIN1, CIN2, CIN3 or ICC at Keio University Hospital and Tsukuba University Hospital from 2012 to 2016. The total cellular DNA was extracted from the recovered cells on a MagNA Pure LC 2.0 (Roche Diagnostic, Indianapolis, IN), and subjected to PCR with PGMY09/11 primers to amplify HPV L1 DNA, followed by reverse blot hybridization for HPV genotyping, as described previously [23]. Based on the genotyping results, DNA samples positive for HPV52 (*n* = 52) or HPV58 (*n* = 47) were selected for subsequent analyses of the whole-genome sequences of HPV52/58. The study protocol was approved by the Ethics Committees at each hospital and the National Institute of Infectious Diseases, and written informed consent for study participation was obtained from each patient.

### Viral whole-genome amplification and next generation sequencing

Full-circle PCR or overlapping PCR was performed with PrimeSTAR® GXL DNA polymerase (Takara, Ohtsu, Japan) to amplify the whole-genome sequences of HPV52/58 as described previously [24]. The sequences of PCR primers were as follows: full-circle PCR for HPV52: HPV52-1758F (5′-ACA CAT ATG GTA ATA GAA CCA CCA AAA-3′) and HPV52-1908R (5′-TAT TGT CAA AGC TAT GCT GTA ATA CTG-3′); overlapping PCR for HPV52: HPV52-1758F and HPV52-5968R (5′-TCC AAG CCT GTA CAG GCC CAC ACC AAC-3′); HPV52-5673F (5′-GTG TAC CTG CCT CCT GTA CCT GTC TCT-3′) and HPV52-1908R; full-circle PCR for HPV58: HPV58-1751F (5′-TAC TAT CAA TTC CTG AAA CAT GTA TGA-3′) and HPV58-1889R (5′-AAT CTA TCT ATC CAT TCT GGT GTT G-3′); overlapping PCR for HPV58: HPV58-1751F and HPV58-5846R (5′-GCC TGA TAC CTT GGG AAC TAA TAC TTT-3′); HPV58-5677F (5′-ACC TGC CTC CTG TGC CTG TGT CTA AGG-3′) and HPV58-1889R. The amplified DNA was separated by agarose gel electrophoresis and purified with the Wizard gel purification kit (Promega, Madison, WI). The purified DNA was converted to a short-fragmented DNA library using the Nextera XT DNA sample prep kit (Illumina, San Diego, CA), followed by size selection with SPRIselect (Beckman Coulter, Brea, CA). The multiplexed libraries were analyzed on a MiSeq sequencer (Illumina) with the MiSeq reagent kit v3 (150 cycle). The complete genome sequences of HPV52/58 were de novo assembled from the total read sequences using the VirusTAP pipeline [25] (https://gph.niid.go.jp/cgi-bin/virustap/index.cgi). The accuracy of the reconstructed whole-genome sequences was verified by read mapping with Burrows-Wheeler Aligner v0.7.12 [26] and subsequent visual inspection by Integrative Genomics Viewer v2.3.90 [27].

### Phylogenetic tree construction

The complete genome sequences of HPV52 (*n* = 52) or HPV58 (*n* = 48) isolates were aligned against each other using MAFFT v7.309 [28] with default parameters, together with the complete genome sequences of HPV52/58 available in GenBank, including HPV52/58 reference genome sequences that represent all variant lineages/sublineages (HPV52: A1, X74481; A2, HQ537739; B1, HQ537740; B2, HQ537743; C1, HQ537744; C2, HQ537746; D, HQ537748; HPV58: A1, D90400; A2, HQ537752; A3, HQ537758; B1, HQ537762; B2, HQ537764; C, HQ537774; D1, HQ537768; D2, HQ537770), and HPV52/58 genome sequences previously determined by us from Japanese CIN1 specimens (HPV52: AB819272, AB819273, AB819274; HPV58: AB819275, AB819276, AB819277, AB819278) [24]. Maximum likelihood trees were constructed using RAxML HPC v8.2.9 [29], employing 1000 bootstrap replicates. Phylogenetic trees were visualized in FigTree v1.4.3.

### Identification of lineage/sublineage-specific SNPs

All the HPV52/58 genome sequences included in the phylogenetic analyses were used to search for viral single nucleotide polymorphisms (SNPs) specific for variant lineages and sublineages. The multiple sequence alignments of the whole-genome sequences of HPV52/58 were sorted according to variant lineage/sublineage and the number of mismatched bases in order to visually differentiate lineage/sublineage-specific SNPs.

### Entropy analysis

Amino acid variations at individual positions of viral proteins were calculated on the basis of Shannon’s equation [30]:

$$ \begin{array}{c}\hfill H(i)=-\sum_{x_i}p\left({x}_i\right){ \log}_2p\left({x}_i\right)\hfill \\ {}\hfill \left({x}_i=\mathrm{G},\mathrm{A},\mathrm{I},\mathrm{V},\dots \dots \right),\hfill \end{array} $$

where *H(i)*, *p(x*_*i*_ *)*, and *i* indicate the amino acid entropy score of a given position, the probability of occurrence of a given amino acid at the position, and the number of positions, respectively. An *H(i)* score of zero indicates absolute conservation, whereas a score of 4.4 indicates complete randomness. The deduced amino acid sequences of eight HPV proteins (E6/E7/E1/E2/E4/E5/L2/L1) of the HPV52/58 isolates were concatenated and aligned with each other using MAFFT. The entropy calculation was performed on the multiple sequence alignments using R v2.11.1 (https://cran.r-project.org) with bio3d package v1.1–6 [31].

### Statistical analysis

All statistical analyses were performed using R v3.3.2. Fisher’s exact test was performed to evaluate a difference in HPV58 A sublineages distribution across histological categories. *P* value <0.05 was regarded as statistically significant. The relative risk for progression from CIN1 to CIN2/3/ICC among HPV58 A sublineages was estimated by calculating adjusted odds ratio with its 95% confidence interval.

## Results

### Study subjects

The study subjects consisted of 52 HPV52-positive cases (CIN1, *n* = 20; CIN2/3, *n* = 21; ICC, *n* = 11), and 47 HPV58-positive cases (CIN1, *n* = 19; CIN2/3, *n* = 20; ICC, *n* = 8). The mean age ± standard deviation of the cases in each histological grade was as follows: for HPV52: CIN1, 38.6 ± 12.0 years; CIN2/3, 38.6 ± 8.3 years; ICC, 59.2 ± 15.7 years; for HPV58: CIN1, 35.6 ± 7.4 years; CIN2/3, 37.1 ± 7.5 years; ICC, 55.8 ± 16.2 years.

### Phylogenetic analysis of HPV52/58 whole-genomes

By performing long-range PCR covering viral whole-genomes followed by next generation sequencing analyses, a total of 100 complete genome sequences of 52 isolates of HPV52 and 48 isolates of HPV58 were obtained from the CIN/ICC cases in Japan (Table 1). The lengths of the determined genome sequences ranged from 7903 to 7982-bp for HPV52, and from 7814 to 7836-bp for HPV58. Nucleotide sequence search for open reading frames (ORFs) identified some deletions and insertions in the viral genes of several HPV52/58 isolates when compared to prototype HPV52/58 genomes (HPV52: X74481; HPV58: D90400) as follows: E2/E4 deletion in three HPV52 isolates (#038, #042, and #043), L2 insertion in one HPV52 isolate (#016), and E1 deletion in one HPV58 isolate (#063). Further, the presence of a premature stop codon was observed in the E4 ORF in one HPV52 isolate (#052) and one HPV58 isolate (#098).

**Table 1:** HPV52/58 genomes obtained in this study

	ID	Histology	HPV type	Age	Length (bp)	Lineage	Accession No.
HPV52	#001	CIN1	52	65	7960	B2	LC270024
	#002	CIN1	52	50	7960	B2	LC270025
	#003	CIN1	52	30	7960	B2	LC270026
	#004	CIN1	51/52	31	7960	B2	LC270027
	#005	CIN1	52/56	24	7960	B2	LC270028
	#006	CIN1	52	47	7960	B2	LC270029
	#007	CIN1	33/52/69	31	7960	B2	LC270030
	#008	CIN1	45/52/53	31	7960	B2	LC270031
	#009	CIN1	16/42/52/53	41	7960	B2	LC270032
	#010	CIN1	52	40	7960	B2	LC270033
	#011	CIN1	42/52/58	23	7960	B2	LC270034
	#012	CIN1	52	64	7960	B2	LC270035
	#013	CIN1	35/52	40	7960	B2	LC270036
	#014	CIN1	52	48	7960	B2	LC270037
	#015	CIN1	52	45	7960	B2	LC270038
	#016	CIN1	52/82	24	7982^**^	B2	LC270039
	#017	CIN1	45/52	31	7960	B2	LC270040
	#018	CIN1	31/52	30	7960	B2	LC270041
	#019	CIN1	52	37	7960	B2	LC270042
	#020	CIN1	33/52	40	7960	B2	LC270043
	#021	CIN2	16/52	31	7960	B2	LC270044
	#022	CIN2	52	36	7960	B2	LC270045
	#023	CIN2	52	28	7960	B2	LC270046
	#024	CIN2	52	41	7960	B2	LC270047
	#025	CIN2	52	39	7960	B2	LC270048
	#026	CIN2	52	53	7960	B2	LC270049
	#027	CIN2	52	30	7960	B2	LC270050
	#028	CIN2	52	40	7960	B2	LC270051
	#029	CIN2	52/58	40	7960	B2	LC270052
	#030	CIN2	52	37	7960	B2	LC270053
	#031	CIN2	52	33	7960	B2	LC270054
	#032	CIN3	18/52/58	36	7960	B2	LC270055
	#033	CIN3	52	42	7960	B2	LC270056
	#034	CIN3	52	48	7960	B2	LC270057
	#035	CIN3	39/52/82	30	7960	B2	LC270058
	#036	CIN3	52	31	7937	A1	LC270059
	#037	CIN3	52	37	7960	B2	LC270060
	#038	CIN3	52	33	7903^*^	B2	LC270061
	#039	CIN3	52	49	7960	B2	LC270062
	#040	CIN3	52	36	7937	A1	LC270063
	#041	CIN3	52	61	7960	B2	LC270064
	#042	ICC (SCC)	52	44	7924^*^	B2	LC270065
	#043	ICC (SCC)	33/39/52	54	7921^*^	B2	LC270066
	#044	ICC (SCC)	52	70	7960	B2	LC270067
	#045	ICC (SCC)	52	68	7960	B2	LC270068
	#046	ICC (Ad)	16/18/52	44	7960	B2	LC270069
	#047	ICC (SCC)	52	74	7960	B2	LC270070
	#048	ICC (SCC)	16/52	47	7960	B2	LC270071
	#049	ICC (SCC)	6/16/52	31	7960	B2	LC270072
	#050	ICC (SCC)	52	76	7960	B2	LC270073
	#051	ICC (SCC)	52	76	7960	B2	LC270074
	#052	ICC (SCC)	52	67	7960	B2	LC270075
HPV58	#053	CIN1	42/58	32	7824	A2	LC270076
	#054	CIN1	58	35	7824	A1	LC270077
	#055	CIN1	58	43	7824	A1	LC270078
	#056	CIN1	58	35	7824	A2	LC270079
	#057	CIN1	58	31	7824	A2	LC270080
	#058	CIN1	16/58/82	27	7824	A1	LC270081
	#059	CIN1	58	36	7824	A2	LC270082
	#060	CIN1	58	48	7836	A3	LC270083
	#061	CIN1	58	34	7824	A1	LC270084
	#062	CIN1	42/52/58	23	7824	A1	LC270085
	#063	CIN1	58	54	7814^***^	A2	LC270086
	#064	CIN1	58	31	7824	A2	LC270087
	#065	CIN1	58/68	44	7824	A1	LC270088
	#066	CIN1	58/68	44	7824	A2	LC270089
	#067	CIN1	58	30	7824	A2	LC270090
	#068	CIN1	58	39	7824	A1	LC270091
	#069	CIN1	58	36	7824	A2	LC270092
	#070	CIN1	16/31/52/58/66	33	7836	A3	LC270093
	#071	CIN1	58	39	7824	A1	LC270094
	#072	CIN1	52/58	34	7824	A2	LC270095
	#073	CIN1	58	27	7824	A1	LC270096
	#074	CIN2	16/52/58	45	7824	A2	LC270097
	#075	CIN2	18/52/58	36	7824	A1	LC270098
	#076	CIN2	53/58/84	23	7824	A1	LC270099
	#077	CIN2	58	45	7824	A2	LC270100
	#078	CIN2	58	32	7824	A1	LC270101
	#079	CIN2	58	56	7823	A2	LC270102
	#080	CIN2	58	45	7823	A1	LC270103
	#081	CIN2	58	38	7824	A2	LC270104
	#082	CIN2	58	40	7820	C	LC270105
	#083	CIN2	58	32	7824	A1	LC270106
	#084	CIN2	58	33	7824	A2	LC270107
	#085	CIN2	58	40	7824	A2	LC270108
	#086	CIN3	58	29	7824	A2	LC270109
	#087	CIN3	58	38	7836	A3	LC270110
	#088	CIN3	58	35	7824	A2	LC270111
	#089	CIN3	58	39	7824	A2	LC270112
	#090	CIN3	58	37	7824	A1	LC270113
	#091	CIN3	58	27	7824	A2	LC270114
	#092	CIN3	58	34	7824	A1	LC270115
	#093	ICC (SCC)	58	77	7824	A2	LC270116
	#094	ICC (Ad)	16/53/58	39	7836	A3	LC270117
	#095	ICC (SCC)	58	79	7824	A2	LC270118
	#096	ICC (SCC)	58	48	7824	A1	LC270119
	#097	ICC (SCC)	58	64	7824	A2	LC270120
	#098	ICC (SCC)	58	54	7824	A1	LC270121
	#099	ICC (SCC)	45/58	36	7824	A2	LC270122
	#100	ICC (SCC)	58	49	7836	A3	LC270123

*CIN* cervical intraepithelial neoplasia, *ICC* invasive cervical cancer, *SCC* squamous cell carcinoma, *Ad* adenocarcinoma. The following sequences were identical: #001, #022 and #033; #004 and #010; #012 and #027; #007 and #018; #020 and #021; #011, #045, #047, and #048; #066, #067, and #084; #058, #068, #075, and #078. *, E2/E4 deletion; **, L2 insertion; ***, E1 deletion

**Fig. 1:**
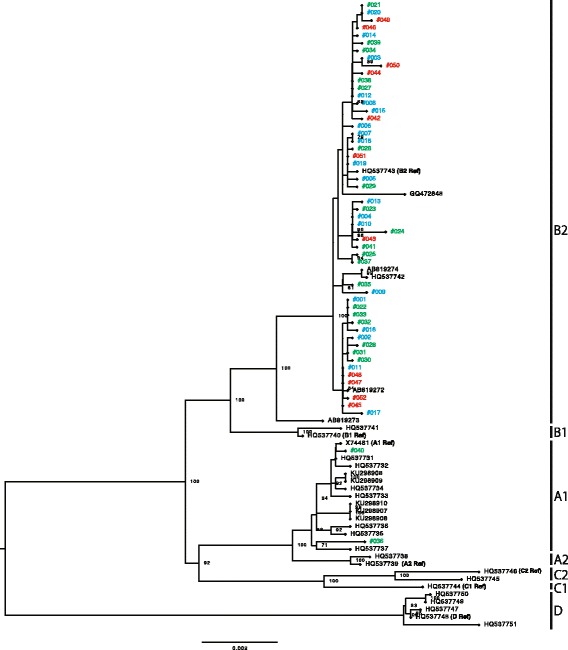
Phylogenetic tree based on the analyses of HPV52 whole-genome sequences of 52 isolates and 31 genomes available from GenBank (total 83 sequences). Phylogenetic analyses were conducted using the Maximum likelihood algorithm by RAxML with 1000 bootstrap replicates. The tree is drawn to scale, with branch lengths measured in the number of substitutions per site. The histological grades of cervical specimens from which the isolates were recovered are shown with colored ID: *blue*, CIN1; *green*, CIN2/3; *red*, ICC

**Fig. 2:**
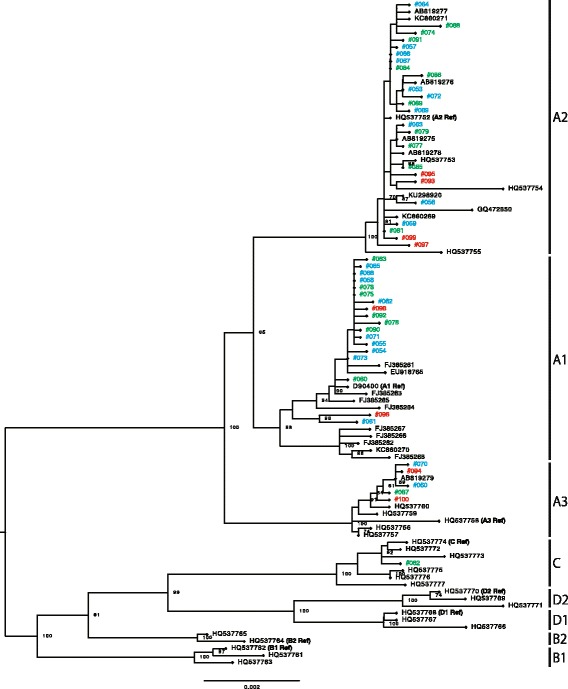
Phylogenetic tree based on the analyses of HPV58 whole-genome sequences of 48 isolates and 46 genomes available from GenBank (total 94 sequences). Phylogenetic analyses were conducted using the Maximum likelihood algorithm by RAxML with 1000 bootstrap replicates. The tree is drawn to scale, with branch lengths measured in the number of substitutions per site. The histological grades of cervical specimens from which the isolates were recovered are shown with colored ID numbers: *blue*, CIN1; *green*, CIN2/3; *red*, ICC

Phylogenetic analyses were conducted with the whole-genome sequences of the HPV52/58 isolates, together with those of reference HPV52/58 genomes that represent individual variant lineages/sublineages. As shown in Fig. 1, several distinct clusters, including each reference genome, confirmed the presence of four lineages and seven sublineages for HPV52. Among the 52 isolates of HPV52 (CIN1, *n* = 20; CIN2/3, *n* = 21; ICC, *n* = 11), 50 isolates belonged to lineage B (sublineage B2) and two isolates belonged to lineage A (sublineage A1). As shown in Fig. 2, the reference genomes of HPV58 consistently revealed the presence of four lineages and eight sublineages. Among the 48 isolates of HPV58 (CIN1, *n* = 21; CIN2/3, *n* = 19; ICC, *n* = 8), 47 isolates belonged to lineage A (sublineage A1, *n* = 18; sublineage A2, *n* = 24; sublineage A3, *n* = 5) and one isolate belonged to lineage C. Interestingly, one HPV58-positive CIN1 specimen yielded two distinct genome sequences of HPV58 (#065 and #066), and these were classified into two different sublineages (A1 and A2), which indicates co-infections with two closely related sublineages of HPV58 in a single patient. The presence of these two sublineages was further confirmed by cloning and Sanger sequencing of HPV58 PCR products obtained from the original DNA sample (data not shown).

Overall, among the Japanese isolates of HPV52/58, the variant distributions were highly biased toward lineage B (sublineage B2) for HPV52 and lineage A for HPV58. The distributions of HPV52/58 variant lineage/sublineage according to cervical histology status when restricted to single infection (HPV52: *n* = 33; HPV58, *n* = 36) are shown in Table 2. Any association of specific lineage/sublineage with a higher risk for CIN2/3 and ICC could not be assessed for the HPV52 isolates, given the dominance of lineage B2 detection across the CIN/ICC cases. The distribution of HPV58 sublineages A1/A2/A3 was almost similar across all histological categories, without a significant difference related to the severity of cervical lesions (Fisher’s exact test, *P* = 0.97). Furthermore, no significant difference in the relative risk for progression from CIN1 to CIN2/3/ICC was observed among HPV58 sublineages A1/A2/A3 (Table 2).

**Table 2:** Distribution of HPV52/58 variant sublineages according to cervical histology status

	Variant	Total	CIN1	CIN2	CIN3	ICC	Adjusted OR^a^ (95% CI)
HPV52	A1	2	0	0	2	0	-
	A2	0	0	0	0	0	-
	B1	0	0	0	0	0	-
	B2	31	9	9	6	7	-
	C	0	0	0	0	0	-
	D	0	0	0	0	0	-
HPV58	A1	13	6	3	2	2	1.0 (reference)
	A2	19	7	5	4	3	1.47 (0.35–6.17)
	A3	3	1	0	1	1	1.71 (0.12–23.9)
	B	0	0	0	0	0	ND
	C	1	0	1	0	0	ND
	D	0	0	0	0	0	ND

Restricted to cases with HPV52 or HPV58 single infection

*OR* odds ratio, *CI* confidence interval, *ND* not determined

^a^relative risk for progression from CIN1 to CIN2/3/ICC compared to HPV58 sublineage A1

### Lineage/sublineage-specific SNPs in HPV52/58 genomes

**Fig. 3:**
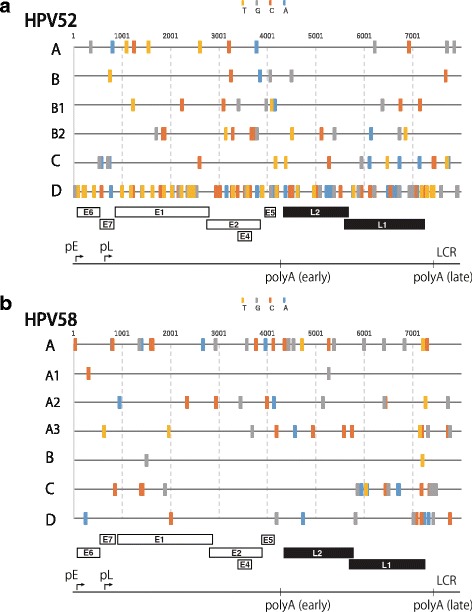
Lineage/sublineage-specific SNPs identified from comparisons of whole-genome sequences of the Japanese HPV52/58 isolates and reference HPV52/58 genomes. The positions of specific SNPs for HPV52 lineages A/B/C/D and sublineages B1/B2 (**a**) and for HPV58 lineages A/B/C/D and sublineages A1/A2/A3 (**b**) are indicated with colored bars. The genome organization of HPV52/58 is shown below: pE, the early promoter; pL, the late promoter; LCR, the long control region; polyA (early) and polyA (late), the early and late polyadenylation signals, respectively

Based on multiple sequence alignments of the complete genome sequences of all HPV52/58 genomes included in the phylogenetic analyses above, SNPs discriminating the variant lineages were extracted from the viral whole-genome sequences. Considering the high prevalence of HPV52 lineage B and HPV58 lineage A in Japan, we also searched for SNPs specific for sublineages of these lineages. All viral SNPs specific for HPV52/58 variant lineages/sublineages found in this study are presented in Fig. 3 and listed in Additional file 1.

For HPV52, as shown in Fig. 3a, many lineage D-specific SNPs were densely distributed throughout the whole genome, which reflects the phylogenetic distance of lineage D from other lineages, as shown in Fig. 1. In contrast, SNPs specific for lineages A/B/C were sparsely distributed compared to lineage D, and those specific for lineages B/C were not found in the E6 region, whereas the E7 region and LCR contained at least one SNP for discriminating each HPV52 variant lineage.

For HPV58, as shown in Fig. 3b, while lineage-specific SNPs for lineage A were dispersed across the viral genome, lineages B/C/D showed relatively biased distributions of such SNPs in the whole-genome sequence. In particular, lineage B had only two diagnostic SNPs in the whole genome, which were positioned in the E1 region and LCR.

### Amino acid variation among the HPV52/58 isolates

**Fig. 4:**
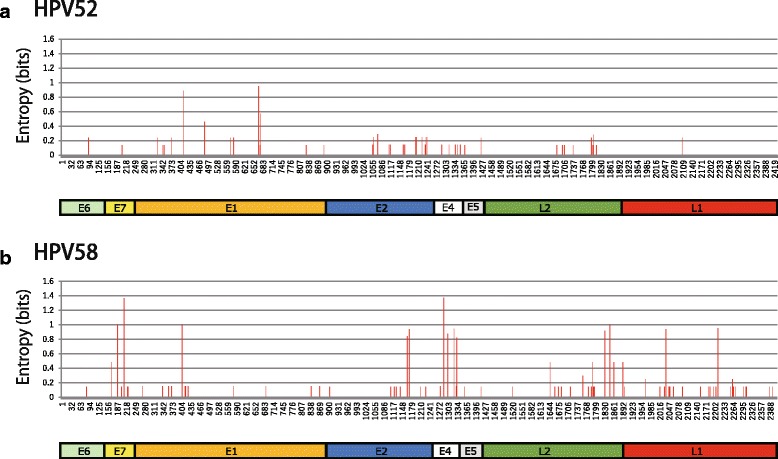
Amino acid variations in viral proteins among the HPV52/58 isolates from Japanese women. Shannon entropy scores representing variations at individual amino acid positions of eight HPV proteins (E6/E7/E1/E2/E4/E5/L2/L1) were calculated using the ORF sequences of the 52 isolates of HPV52 (**a**) and the 48 isolates of HPV58 (**b**). The concatenated viral proteins are shown below

The variability in amino acid sequences of the viral proteins among the HPV52/58 isolates was examined for each genotype by calculating the Shannon entropy scores. As shown in Fig. 4, the overall levels of amino acid variation were apparently lower in HPV52 than in HPV58, which reflects a close relationship among the HPV52 isolates observed as phylogenetic clusters in Fig. 1. Intriguingly, variable amino acid positions were differently distributed across the viral proteins between the HPV52 and HPV58 isolates. In HPV52, the E1 protein showed relatively high variations among the viral proteins, whereas in HPV58, the E7, E4, and L2 proteins showed higher levels of variation in their amino acid sequences than other proteins. The amino acid positions with the top three entropy scores for each genotype were as follows: HPV52: 423 (Lys or Gln) in E1, 168 (Asn or Thr) in E1, and 429 (Ile or Thr) in E1; HPV58: 63 (Asp, Ser, or Gly) in E7, 39 (Trp, Leu, or Ser) in E4, and 41 (Arg or Gly) in E7.

## Discussion

By employing next generation sequencing analyses, we are able to report the largest number of complete genome sequences of HPV52/58 in a single study, and have demonstrated variant distributions of HPV52/58 among Japanese women. Intriguingly, lineage B for HPV52 and lineage A for HPV58 were predominantly detected across the CIN/ICC cases in Japan. Moreover, three HPV52 and four HPV58 genome sequences previously determined by us [24] were also included in HPV52 lineage B and HPV58 lineage A, respectively. These findings are in good agreement with a general trend in HPV52/58 variant distributions, suggesting that HPV52 lineage B and HPV58 lineage A are more prevalent in Asia than in Europe, the Americas, and Africa [16, 32]. Further, a high prevalence of lineage B in HPV52-positive cervical specimens was reported in South Korea [33] and Taiwan [17], and the dominance of lineage A in HPV58-positive specimens was also observed in Taiwan [17], which strongly suggests that such biased distributions of HPV52/58 variant lineages are common among East Asian countries.

In previous studies, a comparison of HPV52/58 variant distributions between different grades of cervical lesions suggested that specific variant lineages, such as HPV52 lineage B [16] and HPV58 lineage A [18], might pose higher risks for cervical cancer development. Meanwhile, because of highly dominant distributions of these HPV52/58 lineages across the CIN/ICC cases in Japan, we were unable to assess a differential risk of these lineages for CIN/ICC progression. Thus, the association of HPV52 lineage B or HPV58 lineage A with cervical cancer development requires further verification with larger sample sizes consisting of mixed distributions of variant lineages.

Previous studies described E7 T20I/G63S substitutions in HPV58 as a high-risk signature for ICC development [19, 34]. Our phylogenetic analysis demonstrated that all sublineage A3 genomes carried this pair of substitutions; 20I (632 T) was specific to sublineage A3, whereas 63S (760A) was not restricted to sublineage A3 but also found in three lineage B genomes (HQ537761, HQ537762, and HQ537763). The T20I/G63S substitutions were observed in five HPV58 isolates in this study, all of which belonged to sublineage A3, although the small sample size precluded our risk assessment of this variation.

The genomic sequences of several HPV52/58 isolates showed some characteristic features, such as deletions in E2/E4 and E1, and insertion in L2. Intriguingly, all E2/E4 deletions observed in the HPV52 isolates were in-frame deletions (36, 39, and 57 nucleotides deletions), and thus supposed to generate internally deleted E2/E4 proteins, which may have altered biological activities for viral transcription, replication and segregation [35]. Of particular interest, these deleted E2/E4 genes were all recovered from CIN3/ICC samples, supporting a prevailing notion that E2 deletion or inactivation favors cervical cancer progression, because the E2 protein generally represses the viral early promoter responsible for E6/E7 expression, which is required for oncogenic transformation of cervical epithelial cells [1]. The E1 deletion in HPV58 and the L2 insertion in HPV52, both observed in CIN1 samples, were not in-frame, and were positioned near their N-terminus. These two genetic changes would be expected to abrogate the functions of the corresponding viral proteins, although the consequence of these deletions on the viral life-cycle and cervical carcinogenesis remains unclear.

By comparing the whole-genome sequences of our Japanese HPV52/58 isolates with those of reference HPV52/58 genomes representing all variant lineages/sublineages, we have presented for the first time a comprehensive list of specific SNPs for discriminating their variant lineages/sublineages. In general, these diagnostic SNPs are dispersed throughout the viral genome, as was reported for HPV16 [36] and HPV6 [37]. However, cautions should be exercised because some genomic regions in HPV52/58 lack such diagnostic SNPs for specific lineage/sublineage identification, as visualized in Fig. 3. Although previous variant classification for HPV52/58 mostly depended on partial sequences in E6, E7, E2, L1 or LCR [17, 20–22], the diagnostic SNPs described in this study will be useful for designing new PCR targets and primers to correctly assign variant lineages/sublineages of HPV52/58 in future epidemiological studies.

Previously, a nucleotide substitution in the L1 region of HPV52 (6764 T to C compared to the prototype, X74481) was reported to generate a mismatched base to the GP6+ primer, one of the consensus primers of GP5+/6+ PCR to amplify L1 DNA of multiple HPV types [38]. Among the 52 isolates of HPV52 obtained in this study, 49 isolates (all sublineage B2) carried this substitution, whereas three isolates (two sublineage A1 and one sublineage B2) had the prototype nucleotide in this position. Since this substitution precluded the detection of HPV52 by GP5+/6+ PCR, leading to underestimation of HPV52 prevalence [38], other PCR methods, such as PGMY09/11 PCR, should be employed for epidemiological surveys on HPV52 in Japan.

The variability of amino acid residues in viral proteins reflects the degree of non-synonymous substitution in the nucleotide sequences of ORFs, and the higher variation at certain amino acid positions suggests that these positions are either neutral or under diversifying selection pressure during viral evolution. From such an evolutionary point of view, the different patterns of amino acid variations in the viral proteins observed in HPV52 and HPV58 are unexpected because these two viruses are genetically closely related to each other and positioned on the same branch of an HPV phylogenetic tree (*Alphapapillomaviruse-9*) [39]. We speculate that different evolutionary pressures may work on the HPV52/58 genomes, thereby restricting or allowing their genomic diversity in a different manner. Subtle differences in the viral life-cycle or virus/host interactions, such as the host immune response, may exist between these closely related HPVs.

An important unanswered question is why HPV52/58 infections are so common in Asia compared to other parts of the world. During the long history of HPV evolution and spread across the globe, these genotypes might have matched a characteristic property of Asian people through as-yet-unknown mechanisms of viral adaptation. Since HPV52/58 infections pose a significant disease burden of cervical cancer on Asian women, further work based on the viral whole-genome sequences, together with elucidation of the genetic background of Asian people including human leukocyte antigen polymorphism, will be required for a better understanding of cervical carcinogenesis driven by these Asia-prevalent HPVs.

## Conclusions

Among the HPV52/58-positive specimens from Japanese women with CIN/ICC, the variant distributions were strongly biased toward lineage B for HPV52 and lineage A for HPV58 across histological categories. Different patterns of amino acid variations were observed in HPV52 and HPV58 across the viral whole-genome.

## Additional file

Additional file 1: Lineage/sublineage-specific SNPs of HPV52/58. (DOCX 53 kb)

## Abbreviations

CIN 1: Cervical Intraepithelial Neoplasia Grade 1
CIN 2: Cervical Intraepithelial Neoplasia Grade 2
CIN 3: Cervical Intraepithelial Neoplasia Grade 3
HPV: Human Papillomavirus
ICC: Invasive Cervical Cancer
LCR: Long Control Region
ORF: Open Reading Frame
SNP: Single Nucleotide Polymorphism
