# Supplementary material for: Whole-genome analysis of human papillomavirus genotypes 52 and 58 isolated from Japanese women with cervical intraepithelial neoplasia and invasive cervical cancer
Source: Infect Agent Cancer. 2017 Aug 4;12:44. doi: 10.1186/s13027-017-0155-4 (PMC5545048; doi:10.1186/s13027-017-0155-4)
Supplement: Additional file 1: — Lineage/sublineage-specific SNPs of HPV52/58. (DOCX 53 kb) [file 13027_2017_155_MOESM1_ESM.docx]

**Lineage/sublineage-specific SNPs of HPV52/58**

________________________________________________________________

Lineage Position SNP Region

________________________________________________________________

HPV52 A 350 G E6

801 A E7

1098 T E1

1244 C E1

1550 T E1

2603 T E1

3204 C E2

3772 A E2

6218 G L1

6917 C L1

7707 G LCR

7856 G LCR

B 751 T E7

3255 C E2

3848 A E2

4055 G E5

4496 G L2

7680 C LCR

B1 1224 T E1

2240 C E1

3087 C E2

3403 G E2/E4

3975 C E5

3977 G E5

4088 T E5

4154 A E5

6375 G L1

6750 C L1

7149 C L1

B2 1703 G E1

1842 C E1

1877 C E1

3141 T E2

3281 C E2

3667 C E2

3675 C E2

3734 C E2

3781 G E2

4508 T L2

5117 C L2

5384 G L2

6138 A L1

6729 G L1

6852 T L1

C 530 G E6

573 A E7

706 G E7

707 A E7

727 G E7

742 A E7

2598 C E1

4171 T -

4376 T L2

5270 C L2

5937 G L1

6111 A L1

6471 T L1

6726 A L1

7140 A L1

7428 C LCR

7449 C LCR

7456 A LCR

7706 T LCR

7728 A LCR

7745 A LCR

D 28 G -

92 C -

97 T E6

200 T E6

237 T E6

425 T E6

573 C E7

766 A E7

1002 T E1

1182 T E1

1222 C E1

1410 G E1

1416 G E1

1422 T E1

1622 T E1

1793 C E1

1799 A E1

1871 T E1

2027 T E1

2042 A E1

2165 C E1

2180 G E1

2225 T E1

2351 G E1

2379 T E1

2439 T E1

2477 T E1

2537 G E1

2937 C E2

3022 C E2

3153 A E2

3268 C E2

3289 T E2

3383 C E2/E4

3533 C E2/E4

3559 G E2/E4

3579 G E2/E4

3584 C E2/E4

3587 T E2/E4

3588 G E2/E4

3639 C E2

3780 A E2

3884 A -

3887 C -

3889 T -

3892 T -

3893 G -

4073 G E5

4088 A E5

4153 A E5

4181 T -

4202 C -

4373 A L2

4463 C L2

4508 C L2

4523 A L2

4616 C L2

4619 T L2

4632 A L2

4955 T L2

4982 A L2

5009 C L2

5082 G L2

5086 A L2

5222 A L2

5261 A L2

5312 A L2

5375 G L2

5507 C L2

5770 A L1

5778 T L1

5880 G L1

5937 C L1

6120 C L1

6201 G L1

6207 C L1

6493 A L1

6543 C L1

6579 A L1

6669 A L1

6721 G L1

6722 A L1

6930 G L1

6945 A L1

6951 G L1

6990 A L1

6993 G L1

7002 C L1

7059 C L1

7089 A L1

7178 T LCR

7259 C LCR

7292 G LCR

7293 T LCR

7393 T LCR

7394 G LCR

7431 T LCR

7578 G LCR

7586 C LCR

7708-7709 N* LCR

7912 G LCR

7928 G LCR

________________________________________________________________

HPV58 A 30 C -

793 A E7

798 C E7

801 C E7

1359 G E1

1396 A E1

1599 C E1

1621 C E1

2669 A E1

2932 G E2

3571 G E2/E4

3571 G E4

3764 C E2

3957 A E5

4123 C -

4354 C L2

4432 G L2

4534 G L2

4711 T L2

5267 C L2

5374 G L2

5994 G L1

6404 G L1

6822 G L1

6828 A L1

7207 T LCR

7296 C LCR

A1 307 C E6

5266 G L2

A2 948 A E1

2340 C E1

2935 C E2

3445 G E2/E4

3988 C E5

4136 A -

5143 G L2

6416 G L1

6434 C L1

7266 T LCR

7726 G LCR

A3 632 T E7

1965 T E1

3685 G E2

4192 C -

4570 A L2

4935 C L2

5579 C L2/L1

5747 C L1

7147 T LCR

7194 C LCR

7316 G LCR

7726 C LCR

7767 G LCR

B 1503 G E1

7200 T LCR

C 852 C E7

1391 C E1

1421 C E1

1884 G E1

5862 G L1

5940 A L1

6039 T L1

6052 A L1

6440 C L1

6441 C L1

6451 C L1

6460 A L1

6497 G L1

6698 A L1

6712 A L1

7185 C LCR

7341 C LCR

7365 G LCR

7427 G LCR

7479 G LCR

D 245 A E6

2010 C E1

4190 G -

4730 A L2

5820 G L1

7011 G L1

7080 C L1

7178 C LCR

7223 A LCR

7234 A LCR

7325 A LCR

7422 G LCR

7771 C LCR

________________________________________________________________

Numbering of nucleotide positions are based on the longest reference genome of each variant lineage: HPV52: A, HQ537739; B, HQ537740; C, HQ537744; D, HQ537748; HPV58: A, HQ537758; B, HQ537764; C, HQ537774; D, HQ537770.
